# Effect of Temperature on Tolbutamide Binding to Glycated Serum Albumin

**DOI:** 10.3390/molecules22040569

**Published:** 2017-03-31

**Authors:** Agnieszka Szkudlarek, Danuta Pentak, Anna Ploch, Jadwiga Pożycka, Małgorzata Maciążek-Jurczyk

**Affiliations:** 1 Department of Physical Pharmacy, School of Pharmacy with the Division of Laboratory Medicine in Sosnowiec, Medical University of Silesia, Jagiellońska 4, 41-200 Sosnowiec, Poland; aniaploch@op.pl (A.P.); jadwiga.pozycka@o2.pl (J.P.); mmaciazek@sum.edu.pl (M.M.-J.); 2 Department of Materials Chemistry and Chemical Technology, Institute of Chemistry, University of Silesia, Szkolna 9, 40-006 Katowice, Poland; danuta.pentak@us.edu.pl

**Keywords:** serum albumin glycation, tolbutamide-albumin binding, fluorescence quenching

## Abstract

Glycation process occurs in protein and becomes more pronounced in diabetes when an increased amount of reducing sugar is present in bloodstream. Glycation of protein may cause conformational changes resulting in the alterations of its binding properties even though they occur at a distance from the binding sites. The changes in protein properties could be related to several pathological consequences such as diabetic and nondiabetic cardiovascular diseases, cataract, renal dysfunction and Alzheimer’s disease. The experiment was designed to test the impact of glycation process on sulfonylurea drug tolbutamide-albumin binding under physiological (T = 309 K) and inflammatory (T = 311 K and T = 313 K) states using fluorescence and UV-VIS spectroscopies. It was found in fluorescence analysis experiments that the modification of serum albumin in tryptophanyl and tyrosyl residues environment may affect the tolbutamide (TB) binding to albumin in subdomain IIA and/or IIIA (Sudlow’s site I and/or II), and also in subdomains IB and IIB. We estimated the binding of tolbutamide to albumin described by a mixed nature of interaction (specific and nonspecific). The association constants $K_{a}$ (L∙mol^−1^) for tolbutamide at its high affinity sites on non-glycated albumin were in the range of 1.98–7.88 × 10^4^ L∙mol^−1^ (λ_ex_ = 275 nm), 1.20–1.64 × 10^4^ L∙mol^−1^ (λ_ex_ = 295 nm) and decreased to 1.24–0.42 × 10^4^ L∙mol^−1^ at λ_ex_ = 275 nm (T = 309 K and T = 311 K) and increased to 2.79 × 10^4^ L∙mol^−1^ at λ_ex_ = 275 nm (T = 313 K) and to 4.43–6.61 × 10^4^ L∙mol^−1^ at λ_ex_ = 295 nm due to the glycation process. Temperature dependence suggests the important role of van der Waals forces and hydrogen bonding in hydrophobic interactions between tolbutamide and both glycated and non-glycated albumin. We concluded that the changes in the environment of TB binding of albumin in subdomain IIA and/or IIIA as well as in subdomains IB and IIB influence on therapeutic effect and therefore the studies of the binding of tolbutamide (in diabetes) to transporting protein under glycation that refers to the modification of a protein are of great importance in pharmacology and biochemistry. This information may lead to the development of more effective drug therapy in people with diabetes.

## 1. Introduction

Serum albumin plays a significant role in drugs pharmacokinetics and can affect pharmacological or toxicity effect of the drug. The drug–serum albumin interaction is an important component in understanding mechanism of action and drugs distribution [1]. The capability of serum albumin to bind aromatic and heterocyclic compounds is largely dependent on the existence of two major binding regions, namely Sudlow’s site I and II which are located within specialized cavities in subdomains IIA and IIIA, respectively [2]. When protein modification induced by physiological or pathological changes occurs, an alteration of the native conformation and efficiency of major binding sites can be expected [3]. Albumin is exposed to numerous structural modifications that can influence its stability, activity and physical and chemical properties, thus affect the functions performed by this protein [4,5]. Major modification concerning serum albumin is non-enzymatic glycation process that begins with the reaction between a reducing sugar and a free amine group on a protein. Glycation leads to the formation of Advanced Glycation End-products (AGEs). AGEs take part in the pathogenesis of many diseases related to aging and also lead to development of many diabetic complications [6]. AGEs exhibit a wide range of chemical structures and thereby have different biological properties some of them are fluorescent like pentosidine and argpyrimidine [7,8]. Based on the literature, Lys-525, 538 and 545 (located near subdomain IIIA), Lys-414, 439, 475, Arg-410, 428, 472 (located in subdomain IIIA) and Lys-199, 212, 233, 276, 281, 286, and Arg-218 (located in subdomain IIA) are the main sites of human serum albumin (HSA) glycation [9,10,11,12,13]. Glucose, fructose, galactose and other reducing sugars can react with proteins through the Maillard reaction (glycation) and form irreversible AGEs [14,15]. Schalkwijk et al. wrote that the formation of AGEs is known to result from the action of various metabolites other than glucose, which are primarily located intracellularly and participate in the non-enzymatic glycation reaction at a much faster rate, such as fructose [16]. They also indicated that despite fructose eightfold higher reactivity, the contribution of extracellular glycation by fructose is considerably less than by glucose because of the low plasma concentration of fructose (5 mmol/L glucose vs. 35 μmol/L fructose). Intracellularly, the level fructose is elevated in many diabetic patients tissues where the polyol pathway is active. Furthermore, in the cells of these tissues, the concentrations of fructose and glucose are of the same magnitude.

**Scheme 1:**
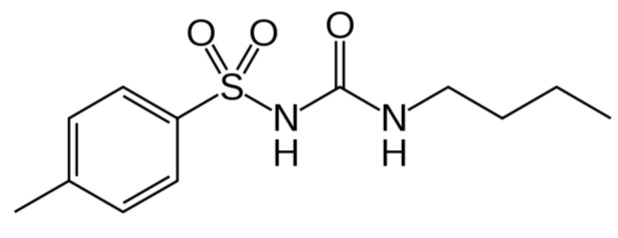
Chemical structure of tolbutamide (TB).

Tolbutamide (1-Butyl-3-(4-methylphenyl)sulfonylurea, TB, Scheme 1) is an oral hypoglycemic drug that was formerly used in a treatment of diabetes type 2. Tolbutamide is metabolized by cytochrome P450 2C9 (CYP2C9) [17]. At therapeutic level TB is strongly bound to plasma protein in 91–96%.

Because the glycation may cause a number of structural changes in the spatial albumin structure that can affect the binding of ligands, the aim of the study was to investigate the influence of glycation process on tolbutamide-albumin binding in temperature corresponding to the human physiological (T = 309 K) and inflammatory states (T = 311 K and T = 313 K) using fluorescence and UV-VIS spectroscopies as a useful tools due to the high sensitivity, rapidity and ease of implementation.

## 2. Results and Discussion

### 2.1. The Structural Modification—Glycation of Serum Albumin

Glycation as a result of the metabolic changes that occur during diabetes causes a number of structural and functional modification in serum albumin. The extent of modification that occurs in protein depends mainly on the time of protein incubation with reducing sugar and a type of reducing sugar. Fructose, as compared with glucose, reacts faster with proteins and produces more protein-bound fluorescence than glucose [14,18].

**Figure 1:**
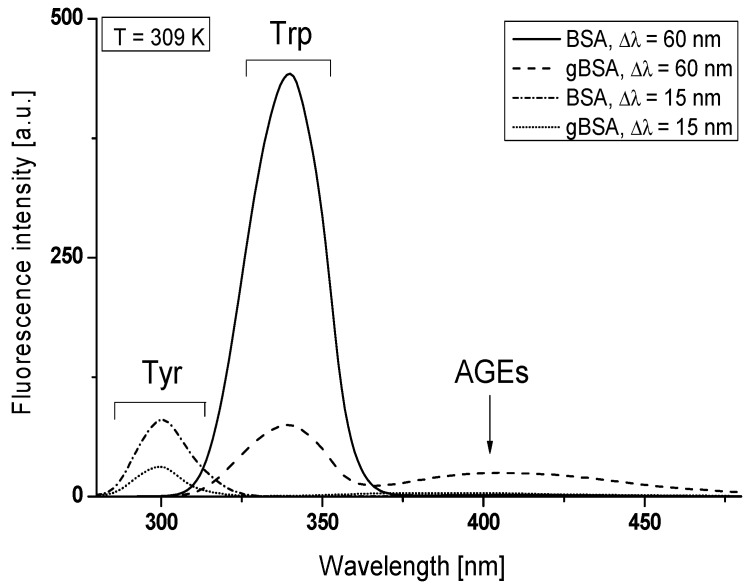
Synchronous fluorescence spectra of non-glycated (BSA: **—** Δλ = 60 nm, -∙-∙-∙ Δλ = 15 nm) and glycated (gBSA: --- Δλ = 60 nm, ∙∙∙ Δλ = 15 nm) bovine serum albumin, BSA, gBSA = 5 × 10^−6^ mol∙L^−1^, T = 309 K.

In the present study, fructose has been used as a bovine serum albumin (BSA) initiator of glycation. In order to focus on the main albumin binding sites, Sudlow’s site I located in subdomain IIA and Sudlow’s site II located in subdomain IIIA, we used λ_ex_ = 275 nm and λ_ex_ = 295 nm excitation wavelengths. λ_ex_ = 275 nm excites both tyrosyl and tryprophanyl residues while λ_ex_ = 295 nm almost exclusively tryptophanyl residue. BSA contains two tryptophanyl residues, Trp-214 (subdomain IIA) and Trp-135 (subdomain IB), whereas BSA tyrosyl residues are located in subdomain IA (Tyr-30), IB (Tyr-138, -140, -148, -150, -156, -157, and -161), IIA (Tyr-263), IIB (Tyr-319, -332, -334, -341, -353, and -370), IIIA (Tyr-401, -411, -452, and -497) [19]. According to the literature, sensitive to in vivo and in vitro glycation are albumin amino acid residues with high nucleophile properties (lysine, Lys and arginine (Arg)) and also a free thiol group of cysteine (Cys-34) located both around and inside of subdomains IIA, IIIA, IB and IA [20]. It means that the glycation process can have an influence on the main Sudlow’s site I and II, and the using of fluorescence quenching method with two excitation wavelengths (275 nm and 295 nm) allows for the analysis of these sites. Moreover, due to the BSA second Trp-135 residue (subdomain IB) the study of glycation impact on the environment of subdomain IB is possible. The influence of glycation process on albumin conformation was investigated by the comparison of synchronous and emission spectra recorded for non-glycated (BSA) and glycated bovine serum albumin (gBSA). Furthermore, the second derivative of fluorescence spectroscopy and spectral parameter A were first used for the identification of subtle changes in the tertiary conformation of proteins. Because the wavelength of λ_ex_ = 295 nm excites almost entirely tryptophanyl residue while λ_ex_ = 275 nm excites both tryptophanyl and tyrosyl residues, and it is impossible to observe separately the fluorescence of tyrosyl residues [21], synchronous fluorescence spectroscopy allows for the separation of the emission spectra originating from the Trp and Tyr residues and more specific information concerning the structure of protein than derived from conventional fluorescence spectroscopy can be obtained. Spectral parameter A has been used in order to monitor the structural changes in tryptophanyl residues microenvironment. According to literature data [22], the synchronous fluorescence spectra were obtained considering the wavelength intervals Δλ = 60 nm and Δλ = 15 nm to evidence the tryptophanyl and tyrosyl residues, respectively (Δλ = λ_em_ − λ_ex_). The synchronous fluorescence spectra of both types of fluorophores in BSA and gBSA at the selected temperature of 309 K have been presented in Figure 1.

**Figure 2:**
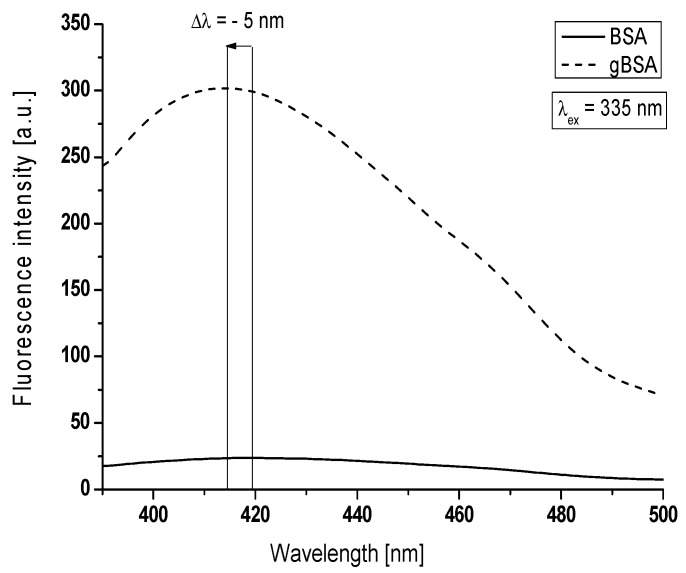
AGEs emission fluorescence spectra of non-glycated and glycated bovine serum albumin at λ_ex_ = 335 nm, BSA, gBSA = 5 × 10^−6^ mol∙L^−1^, T = 309 K.

Synchronous fluorescence spectra of BSA and gBSA Trp and Tyr residues show single emission maxima at λ_max_ = 340 nm and λ_max_ = 300 nm, respectively (Figure 1). It is noteworthy that the synchronous fluorescence spectrum of glycated bovine serum albumin allowed observing an additional signal in the region between λ_em_ = 370 nm and 470 nm with λ_max_ = 405 nm (for the wavelength intervals Δλ = 60 nm) (Figure 1). It can be concluded that this effect is caused by the formation of BSA fluorescence Advanced Glycation End-products (AGEs) and an additional signal derives from the fluorescent AGEs. To prove the impact of glycation by fructose on the formation of AGEs in bovine serum albumin, fluorescence emission spectra of BSA and gBSA were recorded at excitation wavelength of λ_ex_ = 335 nm (absorbance maximum wavelength of AGEs) (Figure 2).

Figure 2 summarizes a typical experiment comparing changes between emission florescence spectra of Advanced Glycation End-products coming from non-glycated and created in glycated albumin. Greater values of maximum fluorescence (F_max_) of AGEs were obtained for glycated albumin (F_414nm_ = 302) than non-glycated (F_419nm_ = 24). It is important to note that a widely accepted assumption is that greater fluorescence intensity is, the more significant protein modification occurs [23]. Argpyrimidine shows fluorescence maximum at about λ_em_ = 400 nm and pentosidine at λ_em_ = 375–385 nm [22]. In opposite to our results, S. Vetter and V. Indurthi reported that Advanced Glycation End-products of human serum albumin (HSA) glycated by glucose do not show typical for pentosidine or argpyrimidine fluorescence but red-shifted fluorescence with maxima about λ_em_ = 420 nm and λ_em_ = 435 nm (excitation at λ_ex_ = 330 nm or λ_ex_ = 365 nm), respectively [24]. The increase in the AGEs fluorescence intensities is accompanied by a blue-shift (Δλ = 5 nm) of maximum fluorescence at λ_ex_ = 335 nm. This blue-shift of BSA maximum fluorescence under the glycation process by fructose makes the changes that AGEs environment becomes more hydrophobic. Rondeau et al. analyzed AGE-products of modified by glucose bovine serum albumin [25]. Using λ_ex_ = 360 nm, they also have observed blue-shifted fluorescence indicating the reduction in the polarity of AGEs environment.

Due to the glycation process fluorescence intensity decreased about 6 times for Trp residues (Δλ = 60 nm) and 2.5 times for Tyr residues (Δλ = 15 nm). The same tendency was also observed at T = 311 K and T = 313 K. These results may suggest a modification of the structure of bovine serum albumin by glycation, especially in tryptophanyl and tyrosyl residues environment, which can affect the main binding sites of albumin in subdomain IIA and/or IIIA (Sudlow’s site I and/or II), and also in subdomains IB and IIB. It is conventional in the fluorescence that the shift of position at maximum emission wavelength (λ_max_) corresponds to changes in polarity around the chromophores molecule. A blue-shift of λ_max_ indicates the increase in hydrophobicity around chromophores while a red-shift of λ_max_ implies increase in hydrophilicity and the chromophores are more exposed to the solvent molecules [22]. Using Δλ = 60 nm and Δλ = 15 nm, no changes in the maximum emission wavelengths on synchronous spectra of BSA and gBSA tryptophanyl and tyrosyl residues were observed (Figure 1) and no changes in the polarity around the indole group of Trp residues have been registered. It seems surprising especially that the maximum emission of Trp is highly sensitive to the local environment.

**Figure 3:**
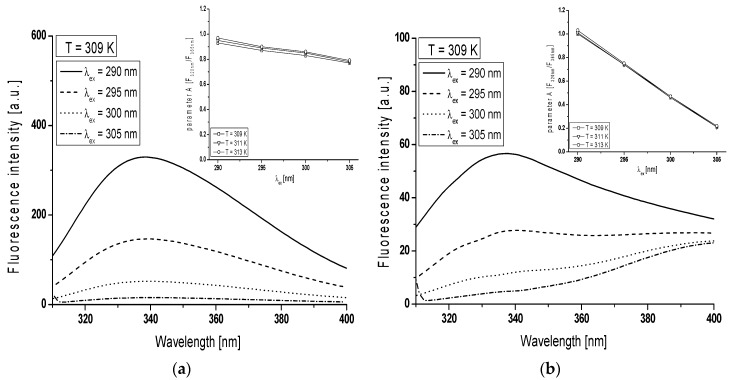
Emission fluorescence spectra of: (**a**) non-glycated (BSA); and (**b**) glycated (gBSA) bovine serum albumin excited at λ_ex_ = 290 nm, λ_ex_ = 295 nm, λ_ex_ = 300 nm and λ_ex_ = 305 nm, T = 309 K. Inserts are plots of parameter $A=\;\frac{F_{320\;nm}}{F_{365\;nm}}$ versus the excitation wavelength at the temperature in the range between 309 K and 313 K for: (**a**) BSA; and (**b**) gBSA.

In order to monitor the structural changes in the Trp microenvironment of BSA (Figure 3a) and gBSA (Figure 3b) tryptophanyl residues (Trp-135 and Trp-214), λ_ex_ from 290 nm to 305 nm has been used and the spectral parameter *A* for BSA and gBSA has been calculated in the range temperature between 309 K and 313 K (Table 1, Figure 3 in the inserts). In order to determine spectral parameter *A* it is necessary to use two wavelengths from the opposite edges of bandwidth, where steepness of the band is large. This causes that for the small change of wavelength the change in the fluorescence intensity is large. In the literature and in the present study the 320 nm and 365 nm wavelengths for proteins containing Trp residues have been used because they correspond to approximately half of the length of band steepness ($A=\;\frac{F_{320\;nm}}{F_{365\;nm}})$. The spectral parameter *A* is the most sensitive indicator of spectral shifts which is less sensitive to experimental errors. As a consequence parameter *A* provides more accurate position of fluorescence spectra in comparison with a position of the maximum fluorescence (λ_max_) [26].

Emission fluorescence spectra of gBSA Trp-135 and -214 residues differ from the Trp residue of non-glycated albumin for all excitation wavelengths (Figure 3). The change in the gBSA spectrum in comparison with BSA is significant especially at λ_ex_ = 305 nm. With the increase of excitation wavelength from 290 nm to 305 nm spectral parameter *A* decreases 1.2 times at all temperatures (Table 1). It probably means that fluorescent spectra of Trp residues shift towards long wavelengths (red-shift). This phenomenon called red-edge excitation shift is caused by microheterogeneity in the tryptophan (Trp) environment and electronic coupling between the tryptophan indole and neighboring dipoles which result in a distribution of electronic transition energies of the Trp [27]. On the other hand, spectral parameter *A* decreases five times the increase of excitation wavelength, obtained for glycated albumin at all temperatures. However, the shape of gBSA Trp residue spectrum (Figure 3b) points out the phenomenon that at the excitation wavelength 365 nm the fluorescence comes probably from AGEs due to the BSA glycation by fructose. It can be concluded that spectral parameter *A* is a tool that confirms the presence of AGEs in BSA-fructose complex regardless of the temperature. At the excitation wavelength λ_ex_ = 305 nm, spectral parameter *A* does not change with the change of temperature (0.21–0.22). This phenomenon means that the increase of temperature does not influence on AGEs concentration.

**Figure 4:**
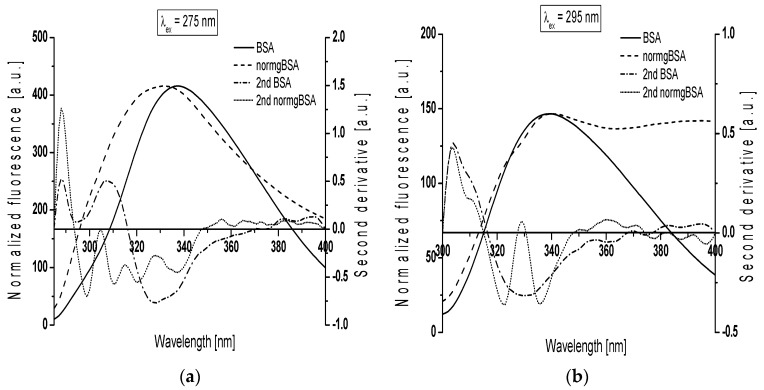
Glycated albumin emission spectrum normalized to non-glycated (normgBSA) and their second derivative fluorescence spectra (2nd BSA, 2nd normgBSA) for: (**a**) λ_ex_ = 275 nm; and (**b**) λ_ex_ = 295 nm. The spectra were normalized to their respective maxima around λ_em_ = 340 nm, T = 309 K.

Another evidence for modification of BSA structure was the analysis of the second derivative spectra. Derivative spectroscopy can extract more information contained within a spectral distribution usually not accessible through the direct spectroscopic measurements and reduce the effect of much spectral interference [28]. Figure 4 presents gBSA emission spectrum (normgBSA) normalized to BSA and their second derivative fluorescence spectra for: (a) λ_ex_ = 275 nm; and (b) λ_ex_ = 295 nm. Before the determination of the second derivative spectra, the fluorescence spectra of gBSA was normalized to respective maxima fluorescence spectra of BSA, λ_em_ = 337 nm and λ_em_ = 339 nm for λ_ex_ = 275 nm and λ_ex_ = 295 nm, respectively.

The changes in the second derivative spectra in the wavelength range 370 nm–400 nm and the range below 320 nm indicate the structure reorganization around tryptophanyl (Trp-214 and Trp-135) and tyrosyl residues (Tyr) in serum albumin, respectively [29,30]. At λ_ex_ = 275 nm, the second derivative fluorescence spectra of non-glycated bovine serum albumin exhibits two peaks maximum (at wavelengths 288 nm and 307 nm) and marked valleys at 295 nm and 328 nm, while the second derivative spectra of normalized glycated bovine serum albumin (normgBSA) has four peaks maximum (288 nm, 305 nm, 315 nm and 328 nm) and four marked valleys (299 nm, 311 nm, 320 nm and 337 nm) (Figure 4a). At λ_ex_ = 295 nm the second derivative fluorescence spectra of BSA exhibits only one peak maximum (at wavelengths 304 nm) with marked valley at 329 nm and small shoulder from the red side of the peak, while the second derivative spectra of normgBSA has two peaks maximum (304 nm and 329 nm), two valleys (322 nm and 336 nm) and also one shoulder from the red side of the peak at wavelengths 304 nm (Figure 4b). Glycation of BSA causes changes in the second derivative fluorescence spectrum at both λ_ex_ = 275 nm and λ_ex_ = 295 nm (Figure 4a,b) indicating that Trp-214, Trp-135 and nineteen tyrosyl residues (Tyr) in bovine serum albumin participate in the process of glycation. The values of the second derivative fluorescence spectra for BSA and normgBSA were determined by peak to peak method (the distance from the positive peak maximum to the negative peak minimum) as an empirical parameter *H* (relative peak composition) defined by Mozo-Villarias [29,30]. The empirical parameter *H* is an indicator of the polarity changes around aromatic residues of proteins. The values of parameter *H* are collected in Table 2.

Glycation of BSA causes the increase in polarity around the tryptophanyl (Trp-214 and Trp-135) (λ_ex_ = 275 nm) and tyrosyl (Tyr) residues (λ_ex_ = 295 nm) that was shown as an increase in the value of parameter *H* (Table 2). Kumar et al. compared the second derivative fluorescence spectra of somatostatin and serum albumin incubated with guanidine hydrochloride. They suggested that first derivative of a signal from the blue-wave spectrum is more sensitive to structural changes [29]. Qualitative analysis indicated that glycation of bovine serum albumin changes (reorganizes) the structure of macromolecule around both tryptophanyl and tyrosyl residues. The main glycation sites are located in the vicinity of known drug binding sites. Wa et al. have characterized the glycation adducts on human serum albumin by matrix-assisted laser desorption/ionization time-of-flight mass spectrometry and found numerous residues of lysines, Lys-12, Lys-51, Lys-199, Lys-205, Lys-439 and Lys-538, to be modified through the formation of fructose-lysine [11]. In addition, some lysine residues of BSA glycated in vitro with glucose have been shown to be involved in the formation of versperlysine products [31]. Other studies revealed Lys-524 as the major glycation site in BSA [32,33] and also Lys-275, Lys-232 and Lys-396-constituted amino acids susceptible to glycated [32,34]. Though less abundant in the amino acid sequence of albumin than lysine residues (23 for 59 lysine residues), arginine residues can also be involved in glycation. The tryptic peptide mapping of modified human serum albumin, in vitro and in vivo, by methylglyoxal, indicated the major modification at Arg-410, which is located in drug binding site II. Minor arginine sites involved in glycation, such as Arg-114, Arg-160, Arg-186, Arg-218 and Arg-428, have also been identified [10]. It is noteworthy that the positions and architecture of fatty acids (FAs) binding sites on serum albumin have been also identified for these sites, in subdomains IB, IIIA, and IIIB [35,36]. Native bovine albumin exhibits two main binding sites for lipoic acid, an effective antioxidant, whereas methylglyoxal-modified protein shows three sequential binding sites with a reduction in affinity for the main one [37] therefore the influence of FAs on glycation process should be taken into account. Yamazaki et al. described strong affinity of fatty acids for albumin via several lysine and arginine sites. They observed the slight effect of oleate, laurate, caproate and linoleate binding on the glycation process [38]. Binding of fatty acids to albumin has an influence on its structure also in terms of binding parameters. Bojko et al. have analysed the influence of fatty acids and on drug-albumin binding [39]. They investigated that the presence of myristic acid alters the affinity between bovine serum albumin and drug.

### 2.2. Effect of Glycation on Binding of Tolbutamide to Bovine Serum Albumin

Hereby, we used BSA in order to prove the glycation of albumin mainly in subdomain IB. The complex between tolbutamide (TB) and non-glycated (BSA) and glycated (gBSA) bovine serum albumin has been studied using the fluorescence emission spectra recorded at the excitation wavelengths λ_ex_ = 275 nm and λ_ex_ = 295 nm, respectively. The wavelength λ_ex_ = 295 nm causes the excitation of two tryptophanyl residues in BSA, i.e., Trp-135 (localized in subdomain IB) and Trp-214 (localized in subdomain IIA) while wavelength λ_ex_ = 275 nm excites not only tryptophanyl residues (Trp-135 and Trp-214), but also tyrosine residues mainly located in subdomains IB (Tyr-138, Tyr-140, Tyr-148, Tyr-150, Tyr-156, Tyr-157, and Tyr-161), IIA (Tyr-263) and IIIA (Tyr-401, Tyr-411, Tyr-452, and Tyr-497) [19]. Consequently, both types of fluorophores and/or their microenvironment may participate in ligand-albumin interaction. Intramolecular interactions of TB with albumin (BSA and gBSA) were studied using the quenching fluorescence method.

**Figure 5:**
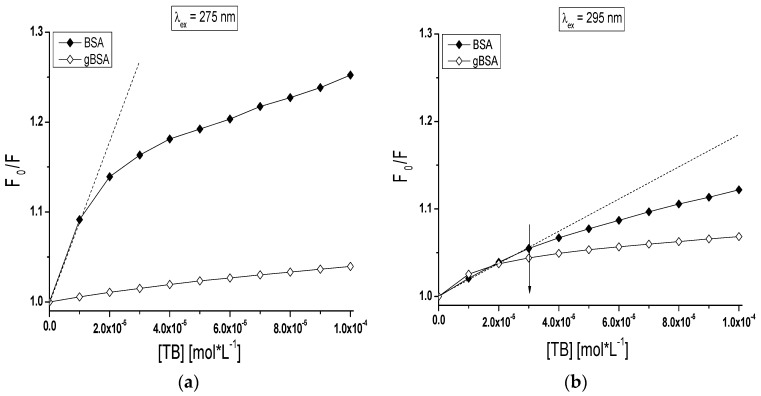
The Stern-Volmer plots of $\frac{F_{0}}{F}$ vs. (TB) (mol∙L^−1^) for TB-BSA (⧫) and TB-gBSA (◊) complex: (**a**) λ_ex_ = 275 nm; and (**b**) λ_ex_ = 295 nm; T = 309 K. The error bars are smaller than the symbols.

Based on the Stern-Volmer plot (Equation (1)), the mode of the interaction between TB and both BSA and gBSA was analyzed (Figure 5). $\frac{F_{0}}{F}$ dependence on TB concentration when only tryptophanyl (Trp-135 and Trp-214) residues have been excited (Figure 5b) displays negative deviation from linearity for both BSA and gBSA. It does mean that there are fluorophores accessible to the quencher (TB) with different value of the Stern-Volmer constant K_SV_ [40]. Trp-135 and Trp-214 are located in different microenvironment therefore the accessibility of TB to these fluorophores also is not the same. Negative deviation of Stern-Volmer plot from linearity allows to estimate that subdomain IIA and IB are involved in TB-serum albumin interaction. However, the linear course of Stern-Volmer plot obtained for gBSA (λ_ex_ = 275 nm) results from the weak quenching of gBSA (~4%) by TB (Figure 5a).

Stern-Volmer curve modified by Lehrer (Equation (2)) was plotted to determine the effective Stern-Volmer constants K_SV_ (L∙mol^−1^) for tolbutamide complexes with BSA and gBSA. The data have been collected in Table 3. The K_SV_ constant determined for TB-gBSA system is higher than that for the TB-BSA system excited at 295 nm for each applied temperature. This phenomenon shows that fructose glycation of BSA changes the fluorophores conformation and makes the tolbutamide diffusion to Trp-135 and Trp-214 easier. The K_SV_ constant determined for TB-gBSA system is smaller than for the TB-BSA system excited at 275 nm in 309 K, 311 K temperature and higher in 313 K. This effect is difficult to explain because using λ_ex_ = 275 nm excitation wavelength excites 22 fluorophores of BSA (20 Tyr and 2 Trp residues).

**Figure 6:**
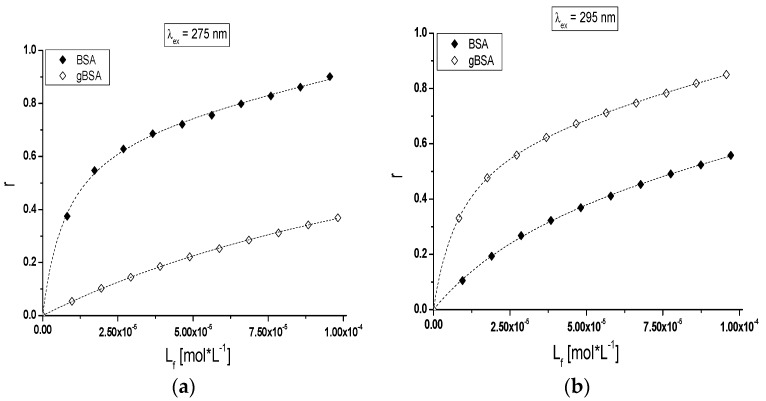
Binding isotherms of non-glycated (BSA,⧫) and glycated (gBSA,◊) bovine serum albumin at 5 × 10^−6^ mol∙L^−1^ concentration with TB at 1 × 10^−5^ mol∙L^−1^–1 × 10^−4^ mol∙L^−1^ concentrations: (**a**) λ_ex_ = 275 nm and (**b**) λ_ex_ = 295 nm; T = 309 K. The error bars are smaller than the symbols.

The analysis of TB interactions with BSA and gBSA excited at λ_ex_ = 275 nm (Figure 6a) and λ_ex_ = 295 nm (Figure 6b) have been presented with the use of a binding isotherm. 

Nonlinear shape of the binding isotherms of BSA and gBSA at both exciting wavelengths (λ_ex_ = 275 nm and λ_ex_ = 295 nm) in the presence of tolbutamide (Figure 6a,b) indicated mixed (specific and nonspecific) nature of TB interaction with both albumins [41].

The quantitative analysis of binding parameters describes the affinity of TB to the binding site. The Klotz Equation (3) allows determining the association constants K_a_ (L∙mol^−1^) and the number of binding sites (n) for the independent classes of TB binding sites in non-glycated and glycated albumin molecule. Only one class of binding site was found for TB in both BSA and gBSA structures (λ_ex_ = 275 nm and λ_ex_ = 295 nm) with the following K_a_ constants values have been calculated: K_a(275 nm)_ (7.88 ± 0.11) × 10^4^ L∙mol^−1^, K_a(295 nm)_ (1.64 ± 0.04) × 10^4^ L∙mol^−1^ and K_a(275 nm)_ (0.42 ± 0.02) × 10^4^ L∙mol^−1^, K_a(295 nm)_ (6.61 ± 0.08) × 10^4^ L∙mol^−1^, respectively. Based on the obtained association constants we can conclude that glycation with fructose changes TB affinity towards bovine albumin. TB has a greater affinity for subdomain IIA and/or IB of glycated albumin than non-glycated protein (Table 3, λ_ex_ = 295 nm). In addition, TB forms stronger complex with non-glycated than glycated albumin (Table 3, λ_ex_ = 275 nm). The differences between the constants calculated for 275 nm and 295 nm are the results of the participation of different excited fluorophores, originating from different subdomains, in the formation of complexes. Moreover, the discrepancy in the values of binding constants calculated at λ_ex_ = 275 nm and λ_ex_ = 295 nm may be explained by greater participation of tyrosyl (λ_ex_ = 275 nm) or only tryptophanyl (λ_ex_ = 295 nm) residues in the complex with tolbutamide. Binding of tolbutamide to non-glycated and glycated serum albumin has been also analyzed by Joseph et al. [42]. The main goal of their study was to examine the binding of tolbutamide to human serum albumin (HSA) at various levels of glycation by the use of high-performance affinity chromatography. They investigated that the binding of tolbutamide to all of tested preparates of glycated human serum albumin (gHSA) could be described by a two site model involving both strong and weak affinity interactions. Joseph et al. reported that the association equilibrium constants $K_{a\;}$ for TB at high affinity sites on gHSA were in the range of 0.8–1.2 × 10^5^ L∙mol^−^^1^ and increased by 1.4-fold in going from normal HSA to mildly gHSA. Moreover, the K_a_ for TB increased by 1.1- to 1.4-fold and by 1.2- to 1.3-fold in going from normal HSA to the gHSA samples at Sudow’s site II and I, respectively. It is noteworthy that we have registered the K_a_ values of the same order (10^4^) despite the use of technique having different fundamentals like fluorescence spectroscopy. Michalcová and Glatz studied the binding of the first generation of sulfonylurea antidiabetics to HSA and its glycated form by capillary electrophoresis-frontal analysis [43]. They reported that the binding constants decreased in the sequence acetohexamide > tolbutamide > chlorpropamide > carbutamide both for normal and glycated human serum albumins, with glycated giving lower values.

### 2.3. The Effect of Temperature on Stability of the TB-BSA and TB-gBSA Complexes

**Figure 7:**
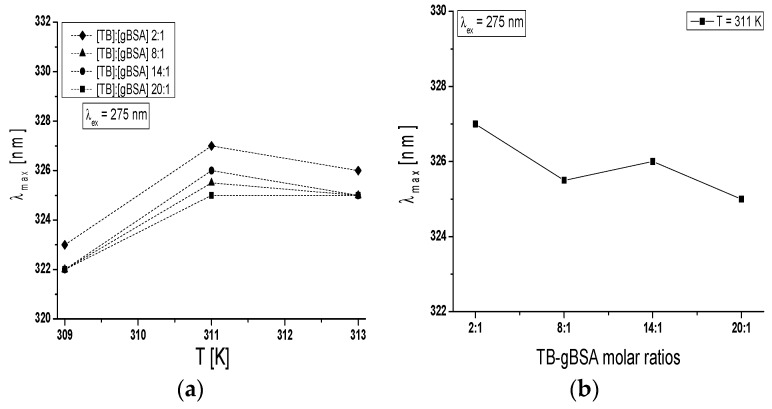
(**a**) Dependence of emission maximum wavelength $(\lambda_{\max})\;$on temperature for TB-gBSA complex; and (**b**) dependence of $\lambda_{\max}\;$ on TB-gBSA molar ratios for T = 311 K; TB:gBSA 2:1, 8:1, 14:1 and 20:1 molar ratios. The error bars are smaller than the symbols.

Maximum wavelength λ_max_ of TB-BSA spectrum at TB:BSA = 2:1; 8:1; 14:1 and 20:1 molar ratios does not depend on temperature in the range between 309 K and 313 K (data not shown). However, the rise of temperature from 309 K to 313 K caused the red-shift of TB-gBSA system maximum wavelength at all studied TB:gBSA molar ratios by 3–4 nm at 275 nm wavelength excitation, when all Tyr and Trp residues potentially fluoresce (Figure 7a) and by 2–3 nm towards blue-shift at 295 nm wavelength excitation, when only Trp residues fluoresce (data not shown). The red-shift of maximum wavelength λ_max_ of TB-gBSA spectrum indicates the increase in fluorophore environment polarity of bovine serum albumin, while the blue-shift of λ_max_ means that amino acid residues (Trps) are located in a more hydrophobic environment and are less exposed to the solvent due to the glycation. Changes in polarity around the fluorophore molecule with the increase in temperature range may be due to the destabilization of the tertiary structure of albumin. Moreover, the formation of TB-BSA and TB-gBSA complexes may affect the microenvironment of binding sites. This could result in the change of non-glycated and glycated bovine albumin sensitivity to temperature. Furthermore, it is interesting to note that at T = 311 K the maximum wavelength (λ_max_) is different for the majority of molar ratios and decreases with the increase of TB:gBSA molar ratio (Figure 7b).

**Figure 8:**
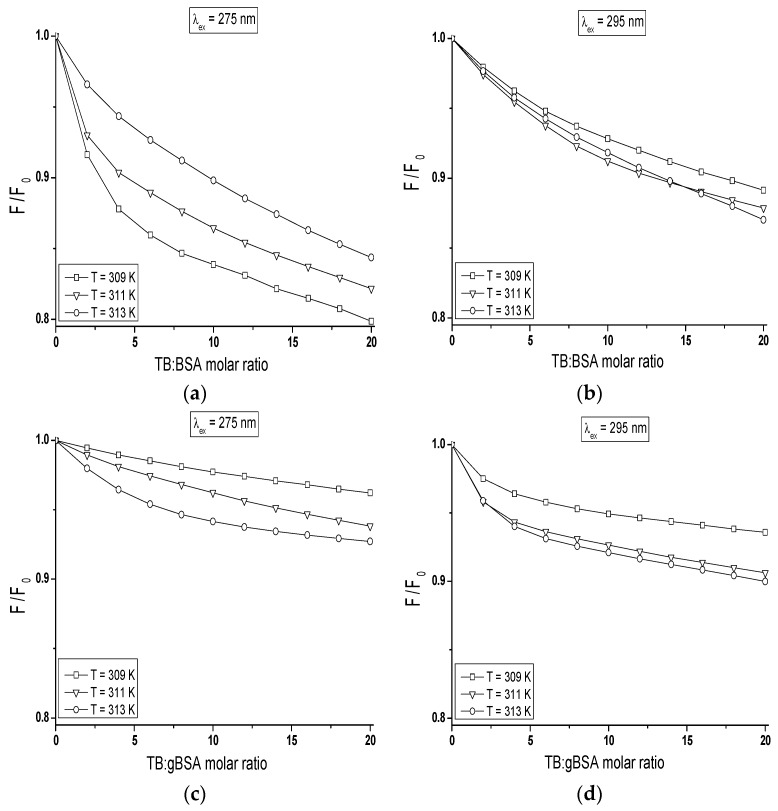
Comparison of the quenching curves of: (**a**,**b**) BSA; and (**c**,**d**) gBSA, in the presence of TB at temperature in the range between 309 K and 313 K; (**a**,**c**) λ_ex_ = 275 nm; and (**b**,**d**) λ_ex_ = 295 nm. The error bars are smaller than the symbols.

The quenching curves of BSA and gBSA fluorescence excited at λ_ex_ = 275 nm and λ_ex_ = 295 nm in the presence of tolbutamide at three different temperatures have been presented in Figure 8.

The course of these curves is temperature dependent and confirms the ability of tolbutamide molecule to receive the energy from excited fluorophores. As it is shown in Figure 8, the fluorescence of BSA and gBSA excited at λ_ex_ = 275 nm is quenched by tolbutamide by 20.1%, 17.8%, 15.6% (Figure 8a) and 3.8%, 6.2%, 7.3% (Figure 8c) for 309 K, 311 K and 313 K, respectively (TB:albumin 20:1 molar ratio). At the same molar ratio, the fluorescence of these two albumins excited at λ_ex_ = 295 nm is quenched by tolbutamide with a lower for TB:BSA and higher for TB:gBSA efficiency, i.e., 10.9%, 12.1%, and 13% (Figure 8b); and 6.4%, 9.4%, and 10% (Figure 8d), respectively. This quenching phenomenon in the temperature range between 309 K and 313 K, more significant for BSA than gBSA (λ_ex_ = 275 nm and λ_ex_ = 295 nm), shows that the energy transfer from non-glycated to tolbutamide is easier than from glycated albumin and confirms the fact of TB-BSA complex formation in close proximity to excited fluorophores. The comparison of the quenching fluorescence curves of both proteins excited at λ_ex_ = 275 nm and λ_ex_ = 295 nm at all applied temperatures shows that fluorescence of BSA is quenched by TB to a greater extend at λ_ex_ = 275 nm than λ_ex_ = 295 nm. For glycated albumin the opposite effect was observed. The fluorescence of gBSA is quenched by TB to a greater extend at λ_ex_ = 295 nm than λ_ex_ = 275 nm. As these wavelengths excite different fluorophores in the albumin, more distinct decrease in fluorescence of protein excited at λ_ex_ = 295 nm indicates that fructose glycated BSA molecule involves preferentially subdomain IIA and/or IB in binding process of tolbutamide. Moreover, the quenching effect of BSA and gBSA fluorescence in the presence of tolbutamide increases (Figure 8b–d) while for BSA excited at λ_ex_ = 275 nm decreases (Figure 8a) with the increase of temperature from 309 K to 313 K. This effect suggests that structural modification of bovine albumin due to glycation causes the changes in the excited fluorophores environment, mainly in the region of tyrosine residues.

**Figure 9:**
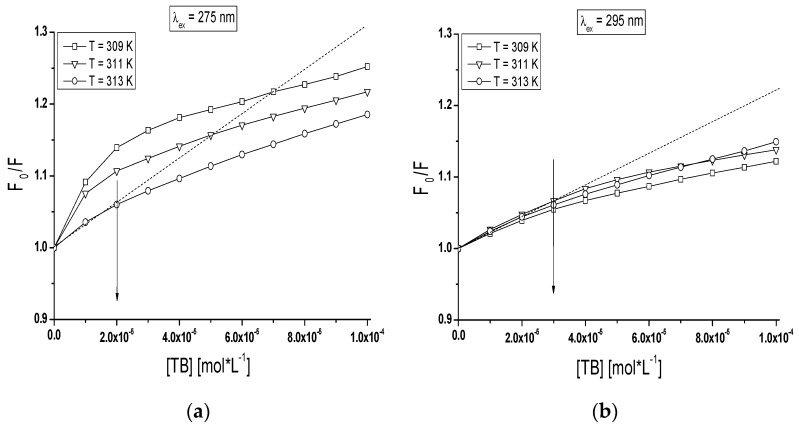

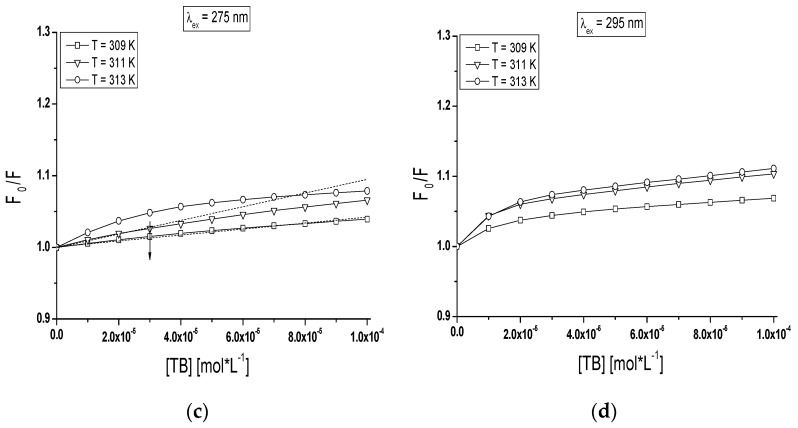
Comparison of the Stern-Volmer curves of $\frac{F_{0}}{F}$ vs. TB concentration (mol∙L^−1^) for: (**a**,**b**) TB-BSA; and (**c**,**d**) TB-gBSA complex in the temperature range between 309 K and 313 K; (**a**,**c**) λ_ex_ = 275 nm; and (**b**,**d**) λ_ex_ = 295 nm. The error bars are smaller than the symbols.

Stern-Volmer analysis is a useful tool for estimation of the accessibility of albumin fluorophores (Trp and Tyr residues) to the drug molecules and for understanding the involved quenching mechanism (dynamic and/or static). Figure 9a,d presents the Stern-Volmer plots for TB-BSA (Figure 9a,b) and TB-gBSA (Figure 9c,d) complexes at λ_ex_ = 275 nm (Figure 9a,c) and λ_ex_ = 295 nm (Figure 9b,d) excitation wavelengths, respectively.

The course of Stern-Volmer curves obtained for BSA and gBSA in the presence of tolbutamide varies depending on the excitation wavelength (λ_ex_ = 275 nm and λ_ex_ = 295 nm) in the temperature range between 309 K and 313 K. It is noteworthy that below TB concentration 3 × 10^−5^ mol∙L^−1^ at all temperatures and at 295 nm excitation wavelength the Stern-Volmer plot showed a linear relationship between quenching effect $\left(\frac{F_{0}}{F}\right)$ of non-glycated BSA and TB concentration (Figure 9b). According to Efting and Ghiron’s theory, it means that Trp-214 and Trp-135 excited state is quenched both by the collisional mechanism and complex formation [40]. A negative deviation from a straight course of Stern-Volmer curves were found for TB-glycated BSA complexes at all applied TB concentration range (Figure 9d). The negative deviation in the Stern-Volmer curve course above TB:BSA and TB:gBSA 4:1 molar ratio points out that both Trp-135 and Trp-214 microenvironment interacts with TB while below TB:BSA and TB:gBSA 4:1 molar ratio a linear course of Stern-Volmer plots (λ_ex_ = 275 nm, T = 309 K and T = 313 K) has been registered (Figure 9a). As for the molar ratio TB:gBSA <6:1 molar ratio a linear course of Stern-Volmer plot at the temperature T = 311 K was observed (Figure 9c). Akbay et al. explained that, during dynamic quenching, the ligand may penetrate the surrounding of albumin molecule and then the fluorescence quenching is caused by the collision of the quencher molecule and fluorophores [44]. However static quenching proves that all excited amino acids residues are equally available for ligand or alternatively selected amino acid residue dominates the fluorescence [45]. The fluorescence quenching process depends on the nature of the fluorophores and the quencher molecule. Our study suggests that glycation of BSA by fructose influences on the tertiary structure of albumin and modifies the fluorescence quenching mechanism of the TB-BSA complex.

Due to general deviation of Stern-Volmer plot from linearity the quenching process was analyzed according to the Stern-Volmer equation modified by Lehrer (Equation (2)) and can be determined by the value of quenching rate constants k_q_ (L∙mol^−1^∙s^−1^) (Equation (1)). The Stern-Volmer constant K_SV_ (L∙mol^−1^) obtained based on the Equation (2) allows to estimate the distance between the excited fluorophore/s and ligand molecule. Reduction in K_SV_ value means the increase in a distance between albumin tryptophanyl and tyrosyl groups and ligand molecule. The Stern-Volmer constant values calculated for TB-BSA and TB-gBSA system at T = 309 K, T = 311 K and T = 313 K temperatures have been collected in Table 3. The decrease in K_SV_ values with the rise of the temperature (from 309 K to 313 K) shows the weakening of tolbutamide accessibility to the tryptophanyl and tyrosyl residues of non-glycated and glycated bovine serum albumin (except TB-gBSA at λ_ex_ = 275 nm). This phenomenon suggests the changes in the protein structure due to the temperature effect, more significant observed at λ_ex_ = 275 nm than at λ_ex_ = 295 nm. Considering the Stern-Volmer constant K_SV_ and the fluorescence lifetime of albumins τ_0_ in the absence of quencher of the order 10^4^ L∙mol^−1^ and 10^−9^ s, respectively, the quenching rate constant k_q_ was of the order 10^13^ L∙mol^−1^∙s^−1^, which largely exceeds the accepted limit of the rate constant of the diffusional quenching implying biopolymers k_q_ = 2 × 10^10^ L∙mol^−1^∙s^−1^ [46]. This observation supports the fact that the experimental quenching of BSA and gBSA fluorescence is due to a predominantly static process resulting in the formation of TB-BSA and TB-gBSA complex.

The association constants K_a_ (L∙mol^−1^) and the number of binding sites n for the independent class of drug binding sites in the bovine albumin have been determined using Klotz Equation (3). The binding parameters (K_a_ and *n*) for TB-BSA and TB-gBSA (λ_ex_ = 275 nm, λ_ex_ = 295 nm) system for specified temperatures were determined and collected in Table 3. Due to the value of determination coefficients larger than 0.99 we concluded that that the interaction between TB and BSA/gBSA is consistent with the site-binding model defined by Klotz Equation (3). The linear Klotz plots (data not shown) at both λ_ex_ = 275 nm and λ_ex_ = 295 nm, at all analyzed temperatures (T = 309 K, T = 311 K and T = 313 K) show that TB occupies in non-glycated albumin only one class of binding sites in BSA subdomain IB or IIA. We observed that glycation of bovine serum albumin did not change the shape of Klotz plots. It means that TB binds to structural altered albumin (glycated BSA) first class of binding sites, similarly as to non-glycated. Based on the variations in association constants K_a_ values (Table 3), the changes in BSA binding ability towards tolbutamide under the glycation by fructose process have been confirmed. Subdomain IIA and IB of fructose glycated BSA has enhanced affinity towards tolbutamide at all applied temperatures than non-glycated BSA. Mentioned subdomains of fructose glycated BSA (λ_ex_ = 275 nm) diminish affinity for tolbutamide. With the increase of temperature, the association constants $K_{a}$ values drop for TB-BSA (λ_ex_ = 275 nm and λ_ex_ = 295 nm) and TB-gBSA (λ_ex_ = 295 nm) complexes and this phenomenon is typical for complex formation. For the TB-gBSA system excited at λ_ex_ = 275 nm, not only K_a_ but also K_SV_ values increase with the increase of temperature. This phenomenon suggests that collisional quenching of gBSA fluorescence dominates the fluorescence quenching than complex formation.

### 2.4. Nature of the Binding Forces for TB-BSA and TB-gBSA Complex Formation

The interaction of ligand and albumin may involve hydrophobic forces, electrostatic interactions, van der Waals interactions and formation of hydrogen bonds. Thermodynamic parameters of interaction, free energy (ΔG), enthalpy (ΔH) and entropy changes (ΔS) are essential to interpret the binding mode. The thermodynamic parameters for the interaction of tolbutamide (TB) with non-glycated (BSA) and glycated (gBSA) albumin have been calculated and collected in Table 4. The negative ΔG values obtained based on the Equation (4) for all TB-BSA and TB-gBSA complexes formed at T = 309 K, T = 311 K and T = 313 K means that in this temperature range the complex tolbutamide-bovine serum albumin forms spontaneously. At the excitation wavelength λ_ex_ = 275 nm the positive values of ΔH and ΔS calculated on the basis of Equations (5) and (6) indicate that hydrophobic forces may play a major role in the binding between TB and gBSA while the negative enthalpy (ΔH) and negative entropy (ΔS) for the interaction between TB and BSA indicate that the hydrogen bonding and van der Waals forces play a major role in the binding process (Table 4). Additionally, involvement of the electrostatic forces usually provides a negative and positive ΔH and ΔS, respectively [47]. At the excitation wavelength λ_ex_ = 295 nm the calculation of ΔH and ΔS was not possible to obtain because the R^2^ = 0.53–0.63 values indicate no “linear” relationship (for straight line regression).

## 3. Materials and Methods

### 3.1. Reagents

Crystallized, lyophilized and high purity bovine serum albumin (BSA, Lot No. 8234H) with fatty acids (fraction V) was purchased from MP Biomedicals LLC (Illkirch, France). Tolbutamide (TB, Lot No. SLBC2764V) was provided by Sigma-Aldrich Chemical Co. (Darmstadt, Germany). d(-)-fructose (FRC, Lot No. A0299881), Tris (hydroxymethyl)aminomethane pure p.a., hydrochloric acid 0.1 mol∙L^−1^ (HCl) were obtained from POCH S.A. (Gliwice, Poland). The membrane filter with 0.20 μm pore size (30 mm in diameter, non-pyrogenic) and methanol were purchased from Merck KGaA (Darmstadt, Germany). All reagents and solvents were of analytical reagent grade.

### 3.2. In Vitro Glycation of BSA

Glycated protein (gBSA) was prepared in vitro by incubation of 5 × 10^−6^ mol∙L^−1^ bovine serum albumin (BSA) in Tris-HCl buffer (0.05 mol∙L^−1^, pH 7.4 ± 0.1) containing 0.015 mol∙L^−1^ sodium azide in the presence of 0.10 mol∙L^−1^ d(-)-fructose (FRC). The reaction mixture was filtered through the membrane filter with 0.20 μm pore size into sterile capped test tubes and incubated at 310 K for 21 days. Control sample of 5 × 10^−6^ mol∙L^−1^ BSA was made from the same stock BSA solution as glycated BSA probe and treated similarly but in the absence of reducing sugar. After incubation the protein stock solutions (control non-glycated and glycated BSA) were extensively overnight dialyzed at room temperature against Tris-HCl buffer with three exchanges to remove residual sugar and again filtered by membrane filter. Fluorescence spectra were recorded 60 min after preparation of the solutions. The pH of protein solution was confirmed with pH meter (FEP20 Metler Toledo). The purity of albumin has been studied and the absorbance at 255 nm and 280 nm ratio was less than 0.5, indicating the high content of protein.

### 3.3. Fluorescence Spectroscopy

From the series of drug-protein interaction study, fluorescence methods are useful tool due to the high sensitivity, rapidity and ease of implementation. These methods allow for examination of BSA interaction with drugs within subdomains IIA and IB. The fluorescence measurements of the samples were recorded in the temperature range between 309 K and 313 K using JASCO fluorescence spectrophotometer FP-6500 equipped with Peltier thermostat. A quartz cuvette with 10 mm path length was used for all fluorescence experiments. The emission fluorescence spectra of non-glycated (BSA) and glycated (gBSA) bovine serum albumin tryptophanyl (Trp) and tyrosyl (Tyr) residues were recorded at the excitation wavelength λ_ex_ = 275 nm while the fluorescence spectra of tryptophanyl residues were measured at λ_ex_ = 295 nm. The measurement range of all emission spectra was recorded from 310 nm to 400 nm and widths of both the excitation and emission slit were set at 3.0 nm. Excitation at λ_ex_ = 335 nm was applied for measurement of the fluorescent Advanced Glycation End-products (AGEs) in BSA and gBSA and for this experiment excitation and emission slit widths were 5.0 nm. The synchronous fluorescence spectra of BSA and gBSA fluorophores were obtained considering the wavelength intervals ∆λ = 15 nm (λ_ex_ = 265 nm–315 nm) and ∆λ = 60 nm (λ_ex_ = 250 nm–440 nm), where ∆λ means the difference between emission (λ_em_) and excitation (λ_ex_) wavelength. Fluorescence second derivative spectra were obtained by the use of Spectra Analysis software (Spectra Manager, version 1.55.00, Jasco Corporation, Easton, MD, USA) using Savitzky and Golay algorithm, second order of polynomial and 15 data points. To obtain the complex TB-BSA and TB-gBSA, the bovine serum albumin solution was titrated using Hamilton syringe directly into the cuvette by the addition of increasing aliquots of TB stock solution (1 × 10^−2^ mol∙L^−1^). Tolbutamide stock solution was dissolved in methanol (not exceeding 1% *v*/*v* in the final concentration). The BSA and gBSA concentration in those experiments was fixed at 5 × 10^−6^ mol∙L^−1^ and the molar ratios TB:BSA and TB:gBSA were varied to 20:1. Light scattering caused by buffer was subtracted from samples fluorescence in each spectrum using software supplied by JASCO (Spectra Manager). Tolbutamide absorbance at the used concentrations was below 0.05; therefore, no correction of inner filter effect in fluorescence spectra has been applied [48].

### 3.4. Calculation of the Stern-Volmer and Association Constants

Using fluorescence data, the quenching curves ($\frac{F}{F_{0}}$ vs. TB:albumin molar ratio) of non-glycated (BSA) and glycated (gBSA) bovine serum albumin in the presence of tolbutamide (TB) have been plotted. The quenching effect of BSA and gBSA fluorescence was analyzed on the basis of the Stern-Volmer equation, which describes ligand movement within the fluorophore microenvironment [46]:

(1) $$\frac{F_{0}}{F}=1+k_{q}\tau_{0}\cdot L=1+K_{\mathrm{SV}}\;\cdot L$$

where $F_{0}$ and F are the fluorescence intensities in the absence and presence of the quencher, respectively; $k_{q}$ is the bimolecular quenching rate constant (L∙mol^−1^∙s^−1^); $\tau_{0}$ is the average fluorescence lifetime of a molecule in the absence of quencher τ_0_ = 6.2 × 10^−9^ s [35]; and K_SV_ is the Stern-Volmer constant (L∙mol^−1^); L is the ligand (TB) concentration (mol∙L^−1^).

To analyze the interactions between the drug and bovine serum albumin (BSA, gBSA) in the high affinity binding sites, the Stern-Volmer (K_SV_) and association (K_a_) constants have been determined. The Stern-Volmer constant K_SV_ was calculated using Stern-Volmer equation modified by Lehrer [49]:

(2) $$\frac{F_{0}}{∆F}=\frac{1}{L}\cdot \frac{1}{f_{a}}\cdot \frac{1}{K_{\mathrm{SV}}}+\frac{1}{f_{a}}$$

where $F_{0}$ and F are the relative fluorescence intensities at fixed wavelength in the absence and presence of the quencher, respectively; ΔF is the difference between F and F_0_; ΔF = F_0_ − F; f_a_ is the fractional maximum fluorescence accessible for the quencher; K_SV_ is the Stern-Volmer constant (L∙mol^−1^); and L is the ligand concentration (mol∙L^−1^).

Association constants K_a_ were calculated by the use of Klotz equation [50]:

(3) $$\frac{1}{r}=\;\frac{1}{n}+\;\frac{1}{n\cdot K_{a}\cdot L_{f}}$$

where $r=\;\frac{L_{b}}{\mathrm{BSA}}$ is the number of ligands moles bound per mole of protein molecule; [L] = [L_b_] + (L_f_), L_b_ and L_f_ are the bound and free drug concentration; BSA is serum albumin concentration (mol∙L^−1^); $L_{b}=\frac{∆F}{∆F_{\max}}\cdot {\mathrm{BSA}}_{\mathrm{total}}$; ΔF_max_ (maximal fluorescence change with complete saturation) is evaluated from the linear part of the $\frac{1}{∆F}$ versus $\frac{1}{L}$; n is the number of binding sites for the independent class of drug binding sites in the albumin molecule; and K_a_ is the association constant (L∙mol^−1^).

Binding isotherms of TB to albumins have been obtained based on the graph of the function *r* = f(L_f_) [51].

### 3.5. Calculation of the Thermodynamic Parameters

Thermodynamic parameters, i.e., free energy (G), enthalpy (H) and entropy changes (S) are essential to characterize ligand (TB)—albumin (BSA, gBSA) interaction. Changes of free energy (ΔG) were calculated from the van’t Hoff equation:

(4) $$∆G=-{\mathrm{RTlnK}}_{a}\;\left[\frac{J}{\mathrm{mol}}\right]$$

where R is the gas constant R = 8.314 J∙(molK)^−1^; T is the experimental temperature (K); and K_a_ is the association constant at corresponding T.

The enthalpy (ΔH) and entropy changes ΔS were calculated from the Equations (5) and (6), respectively:

(5) $$ln\frac{K_{2}}{K_{1}}=\left[\frac{1}{T_{1}}-\frac{1}{T_{2}}\right]\cdot \frac{∆H}{R}$$

where K_1_ and K_2_ are the association constants at T_1_ and T_2_ temperatures, respectively.

(6) ∆G=∆H−T·∆S

### 3.6. Statistics

The mean and relative standard deviation (SD) was calculated from three independent experiments unless otherwise indicated. Points without error bars indicate that SD was less than the size of the symbol.

## 4. Conclusions

Glycation is a general spontaneous process in proteins that has significant impact on their physical and functional properties. These changes in protein properties could be related to several pathological consequences such as diabetic and non-diabetic cardiovascular diseases, cataract, renal dysfunction and Alzheimer’s disease. The work presented in this study can form an important tool in assessing the pharmacological properties of tolbutamide (TB) used in diabetic patients. Based on the fluorescence analyses, it was concluded that: (i) the structure of bovine serum albumin is modified by glycation, especially in tryptophanyl and tyrosyl residues environment which can affect the main binding sites of albumin in subdomain IIA and/or IIIA (Sudlow’s site I and/or II), as well as in subdomains IB and IIB; (ii) glycation of BSA causes the increase in polarity of Trp-214, Trp-135 and Tyr residues environment; (iii) in the interaction of tolbutamide with non-glycated (BSA) and glycated (gBSA) bovine serum albumin, the tryptophanyl and tyrosyl residues take part; (iv) a mixed (specific and nonspecific) nature of TB interaction with BSA and gBSA and only nonspecific with gBSA has been described; (v) the increase in association constants K_a_ (L∙mol^−1^) in TB-gBSA vs. TB-BSA complexes means that in vitro glycated albumin has a higher affinity towards TB in comparison with non-glycated bovine albumin, and the rise of K_a_ values observed for TB-gBSA complex can be explained by the presence of an additional interaction (e.g., electrostatic or hydrogen) as a result of the binding sites conformational changes by glycation; (vi) with the increase of temperature, the association constants K_a_ (L∙mol^−1^) values drop for TB-BSA and TB-gBSA complex besides TB-gBSA (λ_ex_ = 275 nm), which confirms the phenomenon that quenching of BSA fluorescence proceeds via the formation of complex in a ground state; and (vii) temperature dependence suggests the important role of van der Waals forces and hydrogen bonding in hydrophobic interactions between tolbutamide and both glycated and non-glycated albumin.

## Figures and Tables

**Table 1:** The fluorescence intensity of BSA and gBSA at λ_em_ = 320 nm and λ_em_ = 365 nm; λ_ex_ = 290 nm, λ_ex_ = 295 nm, λ_ex_ = 300 nm and λ_ex_ = 305 nm; BSA, gBSA = 5 × 10^−6^ mol∙L^−1^; T = 309 K, T = 311 K and T = 313 K.

Parameter *A*	λ_ex_ = 290 nm	λ_ex_ = 295 nm	λ_ex_ = 300 nm	λ_ex_ = 305 nm
**T = 309 K**				
$\frac{F_{320}}{F_{365}}$ **(BSA) ± SD**	0.93 ± 0.02	0.87 ± 0.02	0.83 ± 0.03	0.77 ± 0.02
$\frac{F_{320}}{F_{365}}$ **(gBSA) ± SD**	1.00 ± 0.01	0.74 ± 0.02	0.46 ± 0.05	0.21 ± 0.06
**T = 311 K**				
$\frac{F_{320}}{F_{365}}$ **(BSA) ± SD**	0.96 ± 0.02	0.89 ± 0.02	0.85 ± 0.02	0.78 ± 0.03
$\frac{F_{320}}{F_{365}}$ **(gBSA) ± SD**	1.02 ± 0.01	0.75 ± 0.02	0.47 ± 0.04	0.21 ± 0.05
**T = 313 K**				
$\frac{F_{320}}{F_{365}}$ **(BSA) ± SD**	0.97 ± 0.01	0.90 ± 0.03	0.86 ± 0.03	0.79 ± 0.04
$\frac{F_{320}}{F_{365}}$ **(gBSA) ± SD**	1.03 ± 0.02	0.75 ± 0.05	0.47 ± 0.04	0.22 ± 0.06

SD = standard deviation.

**Table 2:** Relative peak composition (parameter *H*) of bovine serum albumin residues at λ_ex_ = 295 nm and λ_ex_ = 275 nm.

	*H*_275nm_	λ_min_ (nm)	λ_max_ (nm)	*H*_295nm_	λ_min_ (nm)	λ_max_ (nm)
**BSA**	0.426	295	307	0.018	387	395
**normgBSA**	0.701	299	305	0.023	387	392

**Table 3:** The Stern-Volmer constant of protein fluorescence K_SV_, the association constant K_a_ for the ligand-albumin complex and number of binding sites n determined for TB-BSA and TB-gBSA system; λ_ex_ = 275 nm and λ_ex_ = 295 nm.

TB-BSA						
**T(K)**		**λ_ex_ = 275 nm**			**λ_ex_ = 295 nm**	
	**K_SV_ ± SD (L∙mol^−1^)**	**K_a_ ± SD (L∙mol^−1^)**	***n* ± SD**	**K_SV_ ± SD (L∙mol^−1^)**	**K_a_ ± SD (L∙mol^−1^)**	***n* ± SD**
**309**	(6.04 ± 0.06) × 10^4^	(7.88 ± 0.11) × 10^4^	0.95 ± 0.02	(1.47 ± 0.03) × 10^4^	(1.64 ± 0.04) × 10^4^	1.00 ± 0.02
**311**	(3.99 ± 0.01) × 10^4^	(4.82 ± 0.30) × 10^4^	0.87 ± 0.04	(1.40 ± 0.01) × 10^4^	(1.55 ± 0.02) × 10^4^	0.96 ± 0.01
**313**	(1.78 ± 0.06) × 10^4^	(1.98 ± 0.06) × 10^4^	0.83 ± 0.05	(1.11 ± 0.02) × 10^4^	(1.20 ± 0.02) × 10^4^	0.93 ± 0.03
**TB-gBSA**						
**T(K)**		**λ_ex_ = 275 nm**			**λ_ex_ = 295 nm**	
	**K_SV_ ± SD (L∙mol^−1^)**	**K_a_ ± SD (L∙mol^−1^)**	***n* ± SD**	**K_SV_ ± SD (L∙mol^−1^)**	**K_a_ ± SD (L∙mol^−1^)**	***n* ± SD**
**309**	(0.56 ± 0.01) × 10^4^	(0.42 ± 0.02) × 10^4^	1.09 ± 0.05	(5.22 ± 0.04) × 10^4^	(6.61 ± 0.08) × 10^4^	0.91 ± 0.02
**311**	(1.15 ± 0.06) × 10^4^	(1.24 ± 0.06) × 10^4^	0.88 ± 0.04	(5.02 ± 0.02) × 10^4^	(6.37 ± 0.05) × 10^4^	0.90 ± 0.03
**313**	(2.39 ± 0.03) × 10^4^	(2.79 ± 0.02) × 10^4^	0.85 ± 0.05	(3.69 ± 0.01) × 10^4^	(4.43 ± 0.01) × 10^4^	0.87 ± 0.04

SD = standard deviation.

**Table 4:** The thermodynamic parameters of formation of the TB-BSA and TB-gBSA complex; λ_ex_ = 275 nm and λ_ex_ = 295 nm.

			TB-BSA			
**T (K)**		**λ_ex_ = 275 nm**			**λ_ex_ = 295 nm**	
	**ΔG ± SD** **(kJ∙mol^−1^)**	**ΔH ± SD** **(J∙mol^−1^)**	**ΔS ± SD** **(J∙mol^−1^∙K^−1^)**	**ΔG ± SD** **(kJ∙mol^−1^)**	**ΔH ± SD** **(J∙mol^−1^)**	**ΔS ± SD** **(J∙mol^−1^∙K^−1^)**
**309**	−28.97 ± 0.03			−24.93 ± 0.06		
**311**	−27.88 ± 0.16	−277.69 ± 47.22	−804.21 ± 151.81	−24.95 ± 0.03	---^*)^	---*^)^
**313**	−25.74 ± 0.07			−24.44 ± 0.04		
			**TB-gBSA**			
**T (K)**		**λ_ex_ = 275 nm**			**λ_ex_ = 295 nm**	
	**ΔG ± SD** **(kJ∙mol^−1^)**	**ΔH ± SD** **(J∙mol^−1^)**	**ΔS ± SD** **(J∙mol^−1^∙K^−1^)**	**ΔG ± SD** **(kJ∙mol^−1^)**	**ΔH ± SD** **(J∙mol^−1^)**	**ΔS ± SD** **(J∙mol^−1^∙K^−1^)**
**309**	−24.43 ± 0.12			−28.51 ± 0.03		
**311**	−24.37 ± 0.12	377.87 ± 31.68	1292.58 ± 101.84	−28.60 ± 0.02	---*^)^	---*^)^
**313**	−26.60 ± 0.02			−27.84 ± 0.01		

SD, standard deviation; *^)^, unable to calculate.

## References

[1] Roberts J., Pea F., Lipman J. (2013). The Clinical Relevance of Plasma Protein Binding Changes. Clin. Pharmacokinet..

[2] Sudlow G., Birkett D.J., Wade D.N. (1975). The Characterization of Two Specific Drug Binding Sites on Human Serum Albumin. Mol. Pharmacol..

[3] Oettl K., Stauber R.E. (2007). Physiological and pathological changes in the redox state of human serum albumin critically influence its binding properties. Br. J. Pharmacol..

[4] Shaklai N., Garlick R.L., Bunn H.F. (1984). Nonenzymatic glycosylation of human serum albumin alters its conformation and function. J. Biol. Chem..

[5] Rownicka-Zubik J., Sulkowska A., Bojko B., Maciazek-Jurczyk M., Pozycka J., Pentak D., Sulkowski W.W. (2009). Binding of 6-propyl-2-thiouracil to human serum albumin destabilized by chemical denaturants. J. Photochem. Photobiol. B.

[6] Vlassara H., Bucala R., Striker L. (1994). Pathogenic effects of advanced glycosylation: Biochemical, biologic, and clinical implications for diabetes and aging. Lab. Investig..

[7] Fagugli R.M., Vanholder R., De Smet R., Selvi A., Antolini F., Lameire N., Floridi A., Buoncristiani U. (2001). Advanced glycation end products: Specific fluorescence changes of pentosidine-like compounds during short daily hemodialysis. Int. J. Artif. Organs..

[8] Munch G., Keis R., Wessels A., Riederer P., Bahner U., Heidland A., Niwa T., Lemke H.D., Schinzel R. (1997). Determination of advanced glycation end products in serum by fluorescence spectroscopy and competitive ELISA. Eur. J. Clin. Chem. Clin. Biochem..

[9] Frolov A., Hoffmann R. (2010). Identification andrelative quantification of specific glycation sites in human serum albumin. Anal. Bioanal. Chem..

[10] Ahmed N., Thornalley P.J. (2005). Peptide mapping of human serum albumin modified minimally by methylglyoxal in vitro and in vivo. Ann. N.Y. Acad. Sci..

[11] Wa C., Cerny R.L., Clarke W.A., Hage D.S. (2007). Characterization of glycation adducts on human serum albumin by matrix-assisted laser desorption/ ionization time-of-flight mass spectrometry. Clin. Chim. Acta.

[12] Anguizola J., Matsuda R., Barnaby O.S., Hoy K.S., Wa C., DeBolt E., Koke M., Hage D.S. (2013). Review: Glycation of human serum albumin. Clin. Chim. Acta.

[13] Frost L., Chaudhry M., Bell T., Cohenford M. (2011). In vitro galactosylation ofhuman serum albumin: Analysis of the putative galactosylation site by mass spectrometry. Anal. Biochem..

[14] Szkudlarek A., Sulkowska A., Maciazek-Jurczyk M., Chudzik M., Rownicka-Zubik J. (2016). Effects of non-enzymatic glycation in human serum albumin. Spectroscopic analysis. Spectrochim. Acta A.

[15] Szkudlarek A., Maciazek-Jurczyk M., Chudzik M., Rownicka-Zubik J., Sulkowska A. (2016). Alteration of human serum albumin tertiary structure induced by glycation. Spectroscopic study. Spectrochim. Acta A.

[16] Schalkwijk C.G., Stehouwer C.D., van Hinsbergh V.W. (2004). Fructose-mediated non-enzymatic glycation: Sweet coupling or bad modification. Diabetes Metab. Res. Rev..

[17] Kirchheiner J., Bauer S., Meineke I., Rohde W., Prang V., Meisel Ch., Roots I., Brockmoller J. (2002). Impact of CYP2C9 and CYP2C19 polymorphisms on tolbutamide kinetics and the insulin and glucose response in healthy volunteers. Pharmacogenetics.

[18] Suarez G., Rajaram R., Oronsky A.L., Gaxinowicz M.A. (1989). Nonenzymatic glycation of bovine serum albumin by fructose (fructation). Comparison with the Maillard reaction initiated by glucose. J. Biol. Chem..

[19] Carter D.C., Ho J.X. (1994). Structure of serum Albumin. Adv. Protein Chem..

[20] Rondeau P., Bourdon E. (2011). The glycation of albumin: Structural and functional impacts. Biochimie.

[21] Miller J.N. (1979). Recent advances in molecular luminescence analysis. Proc. Anal. Div. Chem. Soc..

[22] Kessel L., Kalinin S., Nagaraj R., Larsen M., Johansson L. (2002). Time-resolved and steady-state fluorescence spectroscopic studies of the human lens with comparison to argpyrimidine, pentosidine and 3-OH-kynurenine. Photochem. Photobiol..

[23] Furth A. (1988). Methods for assaying nonenzymatic glycosylation. Anal. Biochem..

[24] Vetter S., Indurthi V. (2011). Moderate glycation of serum albumin affects folding, stability, and ligand binding. Clin. Chim. Acta.

[25] Rondeau P., Navarra G., Cacciabaudo F., Leone M., Bourdon E., Militello V. (2010). Thermal aggregation of glycated bovine serum albumin. Biochim. Biophys. Acta.

[26] Maciazek-Jurczyk M., Sulkowska A., Rownicka-Zubik J. (2016). Alteration of methotrexate binding to human serum albumin induced by oxidative stress. Spectroscopic comparative study. Spectrochim. Acta A.

[27] Valeur B. (2002). Molecular Fluorescence. Principles and Applications.

[28] Balestrieri C., Colonna G., Giovane A., Irace G., Servillo L. (1978). Second-Derivative Spectroscopy of Proteins. Eur. J. Biochem..

[29] Kumar V., Sharma V.K., Kalonia D.S. (2005). Second derivative tryptophan fluorescence spectroscopy as a tool to characterize partially unfolded intermediates of proteins. Int. J. Pharm..

[30] Mozo-Villarías A. (2002). Second derivative fluorescence spectroscopy of tryptophan in proteins. J. Biochem. Biophys. Methods.

[31] Nakamura K., Nakazawa Y., Ienaga K. (1997). Acid-stable fluorescent advanced glycation end products: Vesperlysines A, B, and C are formed as crosslinked products in the Maillard reaction between lysine or proteins with glucose. Biochem. Biophys. Res. Commun..

[32] Hinton D.J., Ames J.M. (2006). Site specificity of glycation and carboxymethylation of bovine serum albumin by fructose. Amino Acids.

[33] Wada Y. (1996). Primary sequence and glycation at lysine-548 of bovine serum albumin. J. Mass Spectrom..

[34] Ledesma-Osuna A.I., Ramos-Clamont G., Vazquez-Moreno L. (2008). Characterization of bovine serum albumin glycated with glucose, galactose and lactose. Acta Biochim. Pol..

[35] Curry S., Brick P., Franks N. (1999). Fatty acid binding to human serum albumin: New insights from crystallographic studies. Biochim. Biophys. Acta.

[36] Bhattacharya A.A., Grune T., Curry S. (2000). Crystallographic analysis reveals common modes of binding of medium and long-chain fatty acids to human serum albumin. J. Mol. Biol..

[37] Suji G., Khedkar S.A., Singh S.K., Kishore N., Coutinho E.C., Bhor V.M., Sivakami S. (2008). Binding of lipoic acid induces conformational change and appearance of a new binding site in methylglyoxal modified serum albumin. Protein J..

[38] Yamazaki E., Inagaki M., Kurita O., Inoue T. (2005). Kinetics of fatty acid binding ability of glycated human serum albumin. J. Biosci..

[39] Bojko B., Sulkowska A., Maciazek-Jurczyk M., Rownicka J., Sulkowski W.W. (2010). Influence of myristic acid on furosemide binding to bovine serum albumin. Comparison with furosemide-human serum albumin complex. Spectrochim. Acta A.

[40] Eftink M.R., Ghiron C.A. (1976). Exposure of tryptophanyl residues in proteins. Quantitative determination by fluorescence quenching studies. Biochemistry.

[41] Maciazek-Jurczyk M. (2014). Phenylbutazone and ketoprofen binding to serum albumin. Fluorescence study. Pharmacol. Rep..

[42] Joseph K.S., Anguizola J., Hage D.S. (2011). Binding of tolbutamide to glycated human serum albumin. J. Pharm. Biomed. Anal..

[43] Michalcova L., Glatz Z. (2016). Study on the interactions of sulfonylurea antidiabetic drugs with normal and glycated human serum albumin by capillary electrophoresis-frontal analysis. J. Sep. Sci..

[44] Akbay N., Topkaya D., Ergun Y., Alp S., Gok E. (2010). Fluorescence study on the interaction of bovine serum albumin with two coumarin derivatives. J. Anal. Chem..

[45] Lakowicz J.R. (2006). Principles of Fluorescence Spectroscopy.

[46] Rub M.A., Khan J.M., Asiri A.M., Khan R.H., Din K. (2014). Study on the interaction between amphiphilic drug and bovine serum albumin: A thermodynamic and spectroscopic description. J. Lumin..

[47] Pedley J.B., Naylor R.O., Kirby S.P. (1986). Thermochemical Data of Organic Compounds.

[48] Lakowicz J.R., Weber G. (1973). Quenching of Protein Fluorescence by Oxygen. Detection of Structural Fluctuations in Proteins on the Nanosecond Time Scale. Biochemistry.

[49] Lehrer S.S. (1971). Solute Perturbation of Protein Fluorescence. The Quenching of the Tryptophyl Fluorescence of Model Compounds and of Lysozyme by Iodide Ion. Biochemistry.

[50] Klotz I.M., Hunston D.L. (1971). Properties of graphical representations of multiple classes of binding sites. Biochemistry.

[51] Taira Z., Terada H. (1985). Specific and non-specific ligand binding to serum albumin. Biochem. Pharmacol..

